# Diazido­bis[2,4-diamino-6-(2-pyrid­yl)-1,3,5-triazine-κ^2^ *N* ^1^,*N* ^6^]zinc(II)

**DOI:** 10.1107/S1600536809016055

**Published:** 2009-05-07

**Authors:** Qi-Hua Zhao, Ai-Ling Fan, Li-Nan Li, Ming-Jing Xie

**Affiliations:** a School of Chemical Science and Technology, Key Laboratory of Medicinal Chemistry for Natural Resources, Ministry of Education, Yunnan University, Kunming 650091, People’s Republic of China

## Abstract

In the title mononuclear complex, [Zn(N_3_)_2_(C_8_H_8_N_6_)_2_], the Zn^II^ atom, lying on a twofold rotation axis, is six-coordinated in a distorted octa­hedral environment by four N atoms from two 2,4-diamino-6-(2-pyrid­yl)-1,3,5-triazine ligands and two N atoms from two end-on-coordinated azide ions. N—H⋯N hydrogen bonds between the ligand and azide ion link the complex mol­ecules into a three-dimensional network.

## Related literature

For general background to organic–inorganic hybrid complexes with azide ligands, see: Carranza *et al.* (2008 ▶); Gadad *et al.* (2000 ▶, 2004 ▶); Sun & Du (2005 ▶).
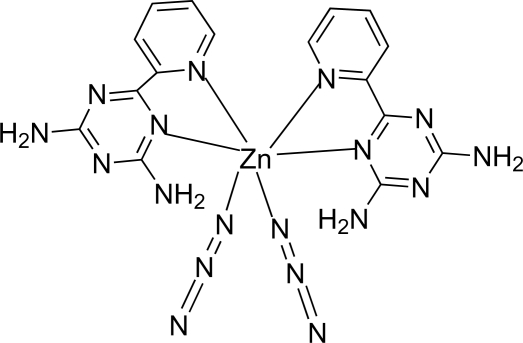


## Experimental

### 

#### Crystal data

[Zn(N_3_)_2_(C_8_H_8_N_6_)_2_]
*M* *_r_* = 525.86
Monoclinic, 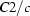

*a* = 18.288 (9) Å
*b* = 14.231 (7) Å
*c* = 9.144 (4) Å
β = 115.382 (5)°
*V* = 2150.2 (18) Å^3^
*Z* = 4
Mo *K*α radiation
μ = 1.19 mm^−1^
*T* = 293 K
0.20 × 0.18 × 0.08 mm

#### Data collection

Bruker APEXII CCD diffractometer
Absorption correction: multi-scan (*SADABS*; Sheldrick, 1996 ▶) *T*_min_ = 0.588, *T*_max_ = 0.841 (expected range = 0.636–0.909)
9145 measured reflections
2569 independent reflections
1766 reflections with *I* > 2σ(*I*)
*R*_int_ = 0.056

#### Refinement

*R*[*F* ^2^ > 2σ(*F* ^2^)] = 0.045
*wR*(*F* ^2^) = 0.111
*S* = 1.00
2569 reflections
159 parameters
H-atom parameters constrained
Δρ_max_ = 0.51 e Å^−3^
Δρ_min_ = −0.41 e Å^−3^

### 

Data collection: *APEX2* (Bruker, 2007 ▶); cell refinement: *SAINT* (Bruker, 2007 ▶); data reduction: *SAINT*; program(s) used to solve structure: *SHELXS97* (Sheldrick, 2008 ▶); program(s) used to refine structure: *SHELXL97* (Sheldrick, 2008 ▶); molecular graphics: *SHELXTL* (Sheldrick, 2008 ▶); software used to prepare material for publication: *SHELXTL*.

## Supplementary Material

Crystal structure: contains datablocks I, global. DOI: 10.1107/S1600536809016055/hy2194sup1.cif

Structure factors: contains datablocks I. DOI: 10.1107/S1600536809016055/hy2194Isup2.hkl

Additional supplementary materials:  crystallographic information; 3D view; checkCIF report

## Figures and Tables

**Table 1:** Selected bond lengths (Å)

Zn1—N7	2.153 (3)
Zn1—N4	2.166 (2)
Zn1—N1	2.202 (2)

**Table 2:** Hydrogen-bond geometry (Å, °)

*D*—H⋯*A*	*D*—H	H⋯*A*	*D*⋯*A*	*D*—H⋯*A*
N5—H5*A*⋯N3^i^	0.86	2.19	3.042 (4)	175
N5—H5*B*⋯N7^ii^	0.86	2.31	3.060 (4)	147
N6—H6*A*⋯N9^iii^	0.86	2.34	3.025 (4)	137
N6—H6*B*⋯N7^iv^	0.86	2.10	2.939 (4)	164

Symmetry codes: (i) 
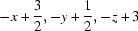
; (ii) 
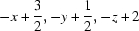
; (iii) 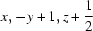; (iv) 
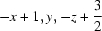
.

**Fig. 1.**
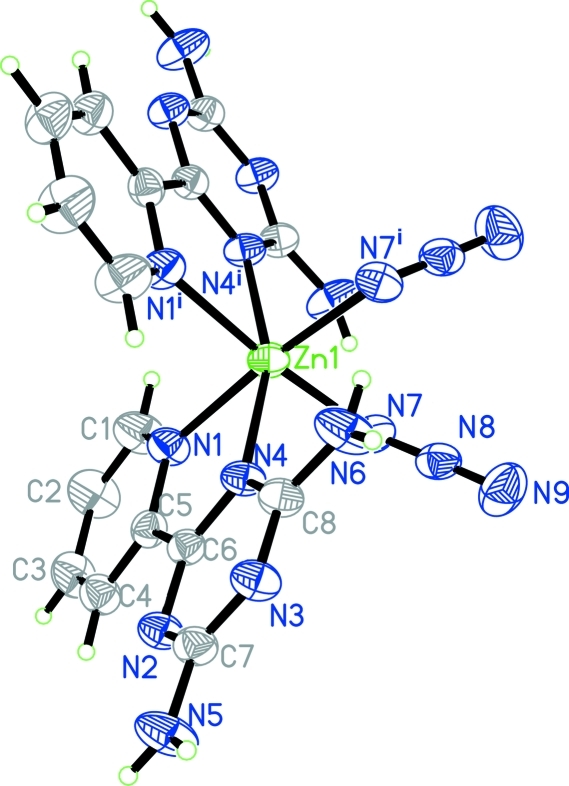
The molecular structure of the title compound. Displacement ellipsoids are drawn at the 30% probability level. [Symmetry code: (i) -x+1, y, -z+3/2.]

**Fig. 2.**
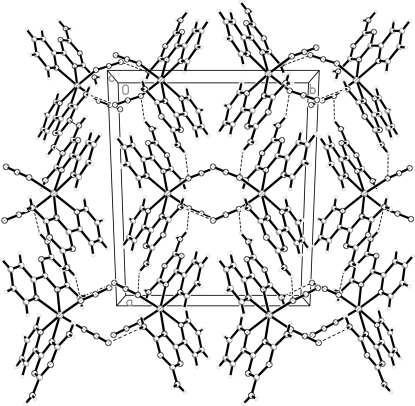
The crystal packing diagram of the title compound, showing hydrogen bonds (dashed lines).

## supplementary crystallographic information

### Comment

At present, the design and synthesis of organic–inorganic hybrid compounds have drawn considerable attention. It was reported that 1*H*-1,3,5-triazole derivatives are a type of heterocyclic compounds with widely biological activity and outstanding capability. Meanwhile, a large number of complexes with azide ligands have been structurally and magnetically characterized (Carranza *et al.*, 2008; Gadad *et al.*, 2000, 2004; Sun & Du, 2005). Herein we report the synthesis and crystal structure of the title compound.

As shown in Fig. 1, the central Zn^II^ atom is coordinated by six N atoms and assumes a distorted octahedral geometry. The Zn—N(amide) bond [2.166 (2) Å] and the Zn—N(azide) bond [2.153 (3) Å] are significantly shorter than the Zn—N(pyridine) bond [2.202 (2) Å] (Table 1). The pyridyl and triazine rings of the ligand are essentially planar. The C3 atom shows an evident deviation of 0.1264 (3)Å from the mean plane through all atoms of the ligand. The two ligands coordinated to the Zn atom form a dihedral angle of 74.80 (7)°. The molecules are connected into a three-dimensional network through N—H···N hydrogen bonds (Table 2 and Fig. 2).

### Experimental

All chemicals used (reagent grade) were commercially available. The compound was synthesized by heating a mixture of Zn(CH_3_COO)_2_ (30 mg, 0.15 mmol), 2,4-diamino-6-pyridyl-l,3,5-triazine (17.6 mg, 0.1 mmol), CH_3_OH (5 ml) and H_2_O (15 ml) at 338 K in stirring condition. After a few minutes, an aqueous solution (2 ml) of sodium azide (6.5 mg, 0.1 mmol) was added to it and stirred for 30 min. Colourless single crystals suitable for X-ray analysis were obtained by slow evaporation at room temperature for three weeks.

### Refinement

H atoms were positioned geometrically and treated as riding on their parent atoms, with N—H = 0.86 and C—H = 0.93 Å and with *U*_iso_(H) = 1.2*U*_eq_(C,N).

### Crystal data

**Table:**

[Zn(N_3_)_2_(C_8_H_8_N_6_)_2_]	*F*(000) = 1072
*M**_r_* = 525.86	*D*_x_ = 1.624 Mg m^−^^3^
Monoclinic, *C*2/*c*	Mo *K*α radiation, λ = 0.71073 Å
Hall symbol: -C 2yc	Cell parameters from 2266 reflections
*a* = 18.288 (9) Å	θ = 1.9–28.6°
*b* = 14.231 (7) Å	µ = 1.19 mm^−^^1^
*c* = 9.144 (4) Å	*T* = 293 K
β = 115.382 (5)°	Block, colourless
*V* = 2150.2 (18) Å^3^	0.20 × 0.18 × 0.08 mm
*Z* = 4	

### Data collection

**Table:**

Bruker APEXII CCD diffractometer	2569 independent reflections
Radiation source: fine-focus sealed tube	1766 reflections with *I* > 2σ(*I*)
graphite	*R*_int_ = 0.056
φ and ω scans	θ_max_ = 28.6°, θ_min_ = 1.9°
Absorption correction: multi-scan (*SADABS*; Sheldrick, 1996)	*h* = −24→24
*T*_min_ = 0.588, *T*_max_ = 0.841	*k* = −18→19
9145 measured reflections	*l* = −12→12

### Refinement

**Table:**

Refinement on *F*^2^	Primary atom site location: structure-invariant direct methods
Least-squares matrix: full	Secondary atom site location: difference Fourier map
*R*[*F*^2^ > 2σ(*F*^2^)] = 0.045	Hydrogen site location: inferred from neighbouring sites
*wR*(*F*^2^) = 0.111	H-atom parameters constrained
*S* = 1.00	*w* = 1/[σ^2^(*F*_o_^2^) + (0.0537*P*)^2^ + 0.3944*P*] where *P* = (*F*_o_^2^ + 2*F*_c_^2^)/3
2569 reflections	(Δ/σ)_max_ < 0.001
159 parameters	Δρ_max_ = 0.51 e Å^−^^3^
0 restraints	Δρ_min_ = −0.41 e Å^−^^3^

### Special details

**Table:**

Geometry. All e.s.d.'s (except the e.s.d. in the dihedral angle between two l.s. planes) are estimated using the full covariance matrix. The cell e.s.d.'s are taken into account individually in the estimation of e.s.d.'s in distances, angles and torsion angles; correlations between e.s.d.'s in cell parameters are only used when they are defined by crystal symmetry. An approximate (isotropic) treatment of cell e.s.d.'s is used for estimating e.s.d.'s involving l.s. planes.

### Fractional atomic coordinates and isotropic or equivalent isotropic displacement parameters (Å^2^)

**Table:**

	*x*	*y*	*z*	*U*_iso_*/*U*_eq_	
Zn1	0.5000	0.26762 (3)	0.7500	0.03110 (17)	
N1	0.57688 (14)	0.16446 (17)	0.7022 (3)	0.0345 (6)	
N2	0.72454 (13)	0.16338 (16)	1.1155 (3)	0.0317 (6)	
N3	0.67379 (15)	0.25546 (16)	1.2722 (3)	0.0335 (6)	
N4	0.59865 (13)	0.24286 (16)	0.9857 (3)	0.0295 (6)	
N5	0.79367 (15)	0.17410 (19)	1.3913 (3)	0.0466 (7)	
H5A	0.7994	0.1940	1.4845	0.056*	
H5B	0.8297	0.1380	1.3842	0.056*	
N6	0.55659 (15)	0.33821 (19)	1.1393 (3)	0.0463 (7)	
H6A	0.5622	0.3608	1.2308	0.056*	
H6B	0.5157	0.3542	1.0517	0.056*	
N7	0.56565 (14)	0.37551 (19)	0.6915 (3)	0.0370 (6)	
N8	0.58831 (15)	0.43984 (19)	0.7856 (3)	0.0372 (6)	
N9	0.61108 (18)	0.5009 (2)	0.8783 (4)	0.0554 (8)	
C1	0.5666 (2)	0.1303 (2)	0.5574 (4)	0.0486 (9)	
H1A	0.5178	0.1421	0.4682	0.058*	
C2	0.6245 (2)	0.0791 (3)	0.5352 (4)	0.0536 (10)	
H2A	0.6149	0.0572	0.4327	0.064*	
C3	0.6968 (2)	0.0602 (2)	0.6659 (4)	0.0452 (8)	
H3A	0.7370	0.0257	0.6537	0.054*	
C4	0.70815 (19)	0.0941 (2)	0.8161 (4)	0.0375 (7)	
H4A	0.7560	0.0818	0.9071	0.045*	
C5	0.64771 (17)	0.14623 (19)	0.8293 (3)	0.0294 (6)	
C6	0.65790 (16)	0.18691 (19)	0.9882 (3)	0.0276 (6)	
C7	0.72914 (17)	0.1992 (2)	1.2585 (3)	0.0320 (7)	
C8	0.61087 (17)	0.2789 (2)	1.1335 (3)	0.0311 (7)	

### Atomic displacement parameters (Å^2^)

**Table:**

	*U*^11^	*U*^22^	*U*^33^	*U*^12^	*U*^13^	*U*^23^
Zn1	0.0264 (3)	0.0377 (3)	0.0228 (3)	0.000	0.00450 (19)	0.000
N1	0.0326 (13)	0.0405 (15)	0.0225 (13)	0.0043 (11)	0.0043 (11)	−0.0033 (10)
N2	0.0282 (13)	0.0402 (15)	0.0218 (13)	0.0042 (10)	0.0060 (10)	0.0004 (10)
N3	0.0307 (13)	0.0454 (16)	0.0206 (12)	0.0050 (11)	0.0075 (10)	0.0004 (10)
N4	0.0244 (12)	0.0399 (15)	0.0198 (12)	0.0021 (10)	0.0052 (10)	−0.0012 (10)
N5	0.0393 (15)	0.070 (2)	0.0202 (13)	0.0202 (14)	0.0027 (11)	−0.0020 (13)
N6	0.0393 (15)	0.070 (2)	0.0212 (13)	0.0195 (14)	0.0050 (12)	−0.0045 (12)
N7	0.0326 (14)	0.0448 (16)	0.0321 (14)	−0.0050 (12)	0.0123 (11)	−0.0025 (12)
N8	0.0285 (14)	0.0446 (17)	0.0338 (15)	−0.0006 (12)	0.0089 (11)	0.0082 (13)
N9	0.061 (2)	0.0489 (18)	0.0433 (17)	−0.0132 (15)	0.0104 (15)	−0.0083 (15)
C1	0.0435 (19)	0.063 (2)	0.0272 (17)	0.0092 (17)	0.0041 (15)	−0.0105 (16)
C2	0.059 (2)	0.065 (2)	0.0317 (18)	0.0114 (19)	0.0149 (17)	−0.0161 (16)
C3	0.051 (2)	0.046 (2)	0.042 (2)	0.0076 (16)	0.0222 (17)	−0.0088 (15)
C4	0.0379 (17)	0.0382 (18)	0.0335 (17)	0.0037 (14)	0.0125 (14)	−0.0006 (14)
C5	0.0316 (15)	0.0283 (15)	0.0265 (15)	−0.0020 (12)	0.0108 (12)	−0.0022 (12)
C6	0.0265 (14)	0.0303 (15)	0.0244 (15)	−0.0014 (12)	0.0094 (12)	0.0012 (12)
C7	0.0291 (15)	0.0372 (17)	0.0246 (15)	0.0008 (13)	0.0066 (12)	0.0047 (12)
C8	0.0284 (15)	0.0384 (17)	0.0231 (15)	0.0021 (13)	0.0078 (12)	0.0020 (12)

### Geometric parameters (Å, °)

**Table:**

Zn1—N7	2.153 (3)	N5—H5B	0.8600
Zn1—N7^i^	2.153 (3)	N6—C8	1.322 (4)
Zn1—N4^i^	2.166 (2)	N6—H6A	0.8600
Zn1—N4	2.166 (2)	N6—H6B	0.8600
Zn1—N1	2.202 (2)	N7—N8	1.202 (4)
Zn1—N1^i^	2.202 (2)	N8—N9	1.159 (4)
N1—C5	1.343 (3)	C1—C2	1.371 (5)
N1—C1	1.346 (4)	C1—H1A	0.9300
N2—C6	1.318 (3)	C2—C3	1.377 (5)
N2—C7	1.372 (4)	C2—H2A	0.9300
N3—C7	1.338 (4)	C3—C4	1.385 (4)
N3—C8	1.339 (4)	C3—H3A	0.9300
N4—C6	1.337 (3)	C4—C5	1.379 (4)
N4—C8	1.371 (4)	C4—H4A	0.9300
N5—C7	1.329 (3)	C5—C6	1.500 (4)
N5—H5A	0.8600		
			
N7—Zn1—N7^i^	89.01 (14)	H6A—N6—H6B	120.0
N7—Zn1—N4^i^	100.61 (9)	N8—N7—Zn1	115.1 (2)
N7^i^—Zn1—N4^i^	92.75 (9)	N9—N8—N7	178.8 (3)
N7—Zn1—N4	92.75 (9)	N1—C1—C2	123.1 (3)
N7^i^—Zn1—N4	100.61 (9)	N1—C1—H1A	118.4
N4^i^—Zn1—N4	161.28 (13)	C2—C1—H1A	118.4
N7—Zn1—N1	87.41 (10)	C1—C2—C3	119.4 (3)
N7^i^—Zn1—N1	174.96 (9)	C1—C2—H2A	120.3
N4^i^—Zn1—N1	91.39 (9)	C3—C2—H2A	120.3
N4—Zn1—N1	76.04 (9)	C2—C3—C4	118.2 (3)
N7—Zn1—N1^i^	174.96 (9)	C2—C3—H3A	120.9
N7^i^—Zn1—N1^i^	87.41 (10)	C4—C3—H3A	120.9
N4^i^—Zn1—N1^i^	76.04 (9)	C5—C4—C3	119.3 (3)
N4—Zn1—N1^i^	91.39 (9)	C5—C4—H4A	120.4
N1—Zn1—N1^i^	96.38 (14)	C3—C4—H4A	120.4
C5—N1—C1	117.3 (3)	N1—C5—C4	122.7 (3)
C5—N1—Zn1	114.71 (18)	N1—C5—C6	115.9 (2)
C1—N1—Zn1	127.1 (2)	C4—C5—C6	121.4 (3)
C6—N2—C7	113.8 (2)	N2—C6—N4	127.0 (2)
C7—N3—C8	115.9 (2)	N2—C6—C5	116.2 (2)
C6—N4—C8	114.7 (2)	N4—C6—C5	116.8 (2)
C6—N4—Zn1	115.90 (17)	N5—C7—N3	119.1 (3)
C8—N4—Zn1	129.38 (19)	N5—C7—N2	116.0 (3)
C7—N5—H5A	120.0	N3—C7—N2	124.9 (2)
C7—N5—H5B	120.0	N6—C8—N3	118.4 (3)
H5A—N5—H5B	120.0	N6—C8—N4	118.1 (2)
C8—N6—H6A	120.0	N3—C8—N4	123.5 (3)
C8—N6—H6B	120.0		

Symmetry codes: (i) −*x*+1, *y*, −*z*+3/2.

### Hydrogen-bond geometry (Å, °)

**Table:**

*D*—H···*A*	*D*—H	H···*A*	*D*···*A*	*D*—H···*A*
N5—H5A···N3^ii^	0.86	2.19	3.042 (4)	175
N5—H5B···N7^iii^	0.86	2.31	3.060 (4)	147
N6—H6A···N9^iv^	0.86	2.34	3.025 (4)	137
N6—H6B···N7^i^	0.86	2.10	2.939 (4)	164

Symmetry codes: (ii) −*x*+3/2, −*y*+1/2, −*z*+3; (iii) −*x*+3/2, −*y*+1/2, −*z*+2; (iv) *x*, −*y*+1, *z*+1/2; (i) −*x*+1, *y*, −*z*+3/2.
